# Recruiting and retaining autistic talent in STEMM

**DOI:** 10.1016/j.isci.2024.109080

**Published:** 2024-02-27

**Authors:** Amber Crabtree, Dave Caudel, Julia Pinette, Chia Vang, Kit Neikirk, Kinuthia Kabugi, Elma Zaganjor, Antentor Hinton

**Affiliations:** 1 Department of Molecular Physiology and Biophysics, Vanderbilt University, Nashville, TN 37232, USA; 2 The Frist Center for Autism and Innovation, Nashville, TN 37212, USA

## Abstract

Autistic adults (AA) have the highest unemployment rate relative to other groups, regardless of disability status. Systemic changes are needed to acquire and retain AA in science, technology, engineering, mathematics, and medicine (STEMM). Here, we discuss the unique challenges AA face in STEMM and possible solutions to overcome them.

## Main text

**Figure:**
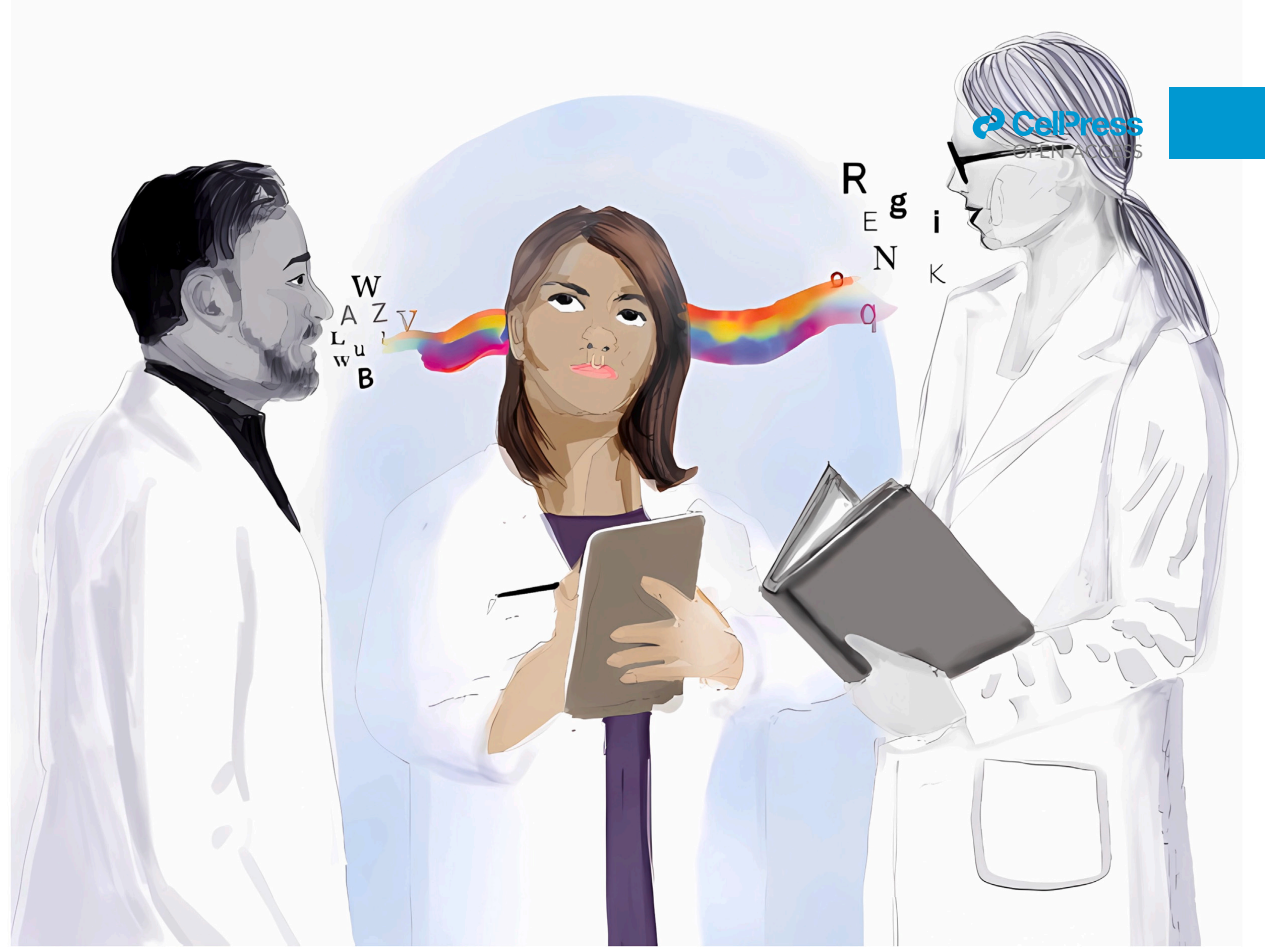
Above image: The invisible isolation that autistic adults in STEMM feel in both conversations and thought processes Failing to account for different ways of thinking and communicating in academia and the workplace can result in interpersonal rifts that leave autistic individuals at a disadvantage and can increase feelings of isolation. Figured created by Neng Vue.

### Introduction

Autism is a type of neurodivergence found in approximately 1 in 36 children in the United States. Compared with the non-autistic population, autistic individuals often experience substantial differences in social interactions, communication, interests, behaviors, thinking, and learning.^1^ The Diagnostic and Statistical Manual of Mental Disorders-5 (DSM-5) characterizes autism as “persistent difficulties in social communication and social interaction,” “restrictive and repetitive patterns of behaviors, activities or interests,” and difficulties with non-verbal communication that are independent of intellectual disability, with several levels of “severity” (See https://www.psychiatry.org/psychiatrists/practice/dsm; Table 1). However, since they are dependent on context, descriptive severity categories should not be used to determine eligibility for and provision of services.

**Table 1:** Important terms related to autism

Term	Meaning
Ableism	Discrimination and social prejudice against disabled people, including defining people by their disabilities and classifying them as inferior to non-disabled people.
Ableist Language	Language that devalues individuals with disabilities or otherwise suggests that people with disabilities are “broken” or “defective” humans. May take the form of subtle jokes or euphemisms.
ADA	Americans with Disabilities Act; an overarching framework that includes accommodations to ensure that disabled individuals have equal opportunities compared with non-disabled individuals under the in the United States (see https://www.ada.gov). If an autistic adult discloses their disability to receive accommodation, faculty and employers must follow the reasonable accomodations listed from a disability/access office.
Autistic	A commonly accepted way to refer to individuals diagnosed with autism. (See https://autisticnotweird.com/autismsurvey/ regarding a large survey that asked autistic individuals how they speak about autism.) However, other studies have shown that Dutch individuals prefer person-first language (see https://journals.sagepub.com/doi/full/10.1177/13623613221117914). This article uses identity-first language through the acronym autistic adults (AA) as it is preferential for the autistic first-author, but we note there may be individual differences in preference between autistic individuals.
Autism	An invisible disability or neurological divergence characterized by differences in social interactions and communication along with restricted interests and repetitive behaviors compared with the non-autistic population. Also referred to as autism spectrum disorder outside of most autistic groups. Autism currently has three severity levels (listed below), as defined in the DSM-5, although these severity levels have been criticized.
Autism/ASD-Level 1	“Requiring support.”
Autism/ASD-Level 2	“Requiring substantial support.”
Autism/ASD-Level 3	“Requiring very substantial support.”
United Nations Convention on the Rights of Persons with Disabilities and its Optional Protocol	Over 180 countries have ratified the United Nations Convention on the Rights of Persons with Disabilities and its Optional Protocol, which offers similar protections as the ADA (see https://social.desa.un.org/issues/disability/crpd/convention-on-the-rights-of-persons-with-disabilities-crpd).

As previously discussed in a compelling career feature from Nature (see https://www.nature.com/articles/d41586-022-04248-5), many neurodivergent individuals in science can feel ostracized and in lack of appropriate support. This article seeks to expand upon this concept, detailing, technology, engineering, mathematics, and medicine (STEMM), including the unique challenges they face and possible solutions to overcome them.

### Current barriers

The autistic population currently has the highest unemployment rate compared with all other groups, regardless of disability status.^2^ An Australian study found that individuals with disabilities other than autism were twice as likely as autistic individuals to have a 4-year degree or higher. Moreover, groups without disabilities were over four times more likely than autistic individuals to attain higher education.^2^ The same study found that 28.7% of the general population without disabilities had some level of higher education compared with 6.5% of the autistic population.^2^ A US study estimated that 40% of AA are employed, though only 15% were estimated to have full-time employment.^3^

Still, AA are more likely to major in STEMM subjects compared with other disabled adults and the general population: approximately 30% of autistic students major in STEMM.^4^ Of these individuals, many are male and white, both of which are characteristics that aid in college persistence.^5^ Large-scale studies similarly show that the vast majority of AA (approx. 80%) attend a community college at some point in their college career, and STEMM fields are a positive retention factor in postsecondary education for autistic students.^5^

Nevertheless, multiple issues, including a lack of belongingness, can arise for autistic college students^6^ and account for the observed varying outcomes in STEMM. Over 50% of autistic college students report that they have significant trouble making conversation or cannot converse.^4^ Routine and consistency are necessary for many AA, and a lack of such routine can derail them and potentially harm their persistence.^4^ Past analyses have shown that autistic individuals often have decreased cognitive flexibility, phonemic fluency, and working memory in comparison to non-autistic people,^7^ which can cause difficulties in maintaining college attendance and/or careers. These data underscore the dire need for inclusive systemic changes to acquire and retain AA in STEMM.

### The autistic advantage: A strengths-based employment approach

Often, autism is considered from a deficit model, which continues to influence the experimental design of many studies, excluding the participation and characteristics of autistic individuals in STEMM (see https://www.psychologicalscience.org/observer/gs-navigating-academia-as-neurodivergent-researchers). Studies often discuss the challenges that autistic individuals face while overlooking their unique strengths.^7^ This is alarming, especially considering that many autistic strengths such as having both autonomous and lateral thinking can be very beneficial in STEMM fields.^2^ Some additional strengths include creativity with a unique perspective and an ability to maintain intense focus on tasks when given sufficient support. Despite the limited literature on the topic, systematic reviews have highlighted that autistic individuals tend to show increased attention to detail and certain heightened skills, including memory, visuospatial ability, and computation.^6^ This can translate to a perfectionist attitude that ensures high-quality work, even when the task is repetitive.^6^ This does not mean autistic individuals should only be relegated to repetitive jobs; rather, AA should be given the chance to thrive, such as technology-related roles, which may be facilitated by a markedly common high work ethic in AA.^6^ Furthermore, meta-analyses show AA may have better planning, decision-making skills, and semantic verbal fluency than non-AA.^7^ There is tremendous heterogeneity in the autistic population in general and in terms of executive functioning capabilities, necessitating different support systems, and some AA may not experience large executive function deficits.^8^ Though autistic individuals experience difficulties in employment,^3^ their unique advantages can be helpful in the context of STEMM, but greater support and a better understanding of autistic strengths and limitations are necessary. Comprehensively, Jones (2023) recently interviewed 37 autistic academics to summarize current positive aspects and challenges within academia.^9^ Here, we further elaborate on autistic-related challenges in STEMM and offer potential solutions or areas for improvement.

### Challenges and improvements

“I’m not disabled by my autism; I’m disabled by my environment.”—Dr. Jac Den Houting, Psychologist and autistic activist.

#### Disclosure and Diagnosis

##### Challenge

A major challenge for AA is the decision to disclose their disability.^1^ This decision can feel especially daunting for individuals whose disability is invisible as they may feel the need to justify their need for accommodations.^1^ However, it should be noted that those who choose not to disclose their disability status can face isolation, which can increase their chances of falling out of the STEMM pipeline.^1^ Individuals who have not received an official diagnosis can still disclose their disability to colleagues, but they may not be guaranteed protection under the Americans with Disabilities Act (ADA).^1^ Nevertheless, a DSM-5 diagnosis is imperfect and often expensive which can significantly affect diagnosis accessibility.

##### Solutions

When considering disclosure, AA must weigh the potential benefits and risks and remember that accommodations exist to ensure that disabled individuals have equal opportunities under the ADA and other governing bodies (Table 1). Yet, given the challenges associated with diagnosis and disclosure, we must shift away from systemic stigmatization of disclosure. We believe that professional and educational institutions must generate a positive feedback loop in which AAs are increasingly retained in STEMM fields, thereby encouraging other autistic individuals to enter STEMM fields. Adopting an empathetic and supportive approach that applies to all individuals, regardless of whether we know them to be autistic, will benefit everyone.

#### Communication differences

##### Challenge

Interpersonal misunderstandings can become problematic when the invisible differences that make someone autistic are forgotten. Over 50% of AA feel they have significant difficulties with communication.^4^ This can have a notable impact in STEMM fields, where effective communication is vital both to the scientific and non-scientific communities. One of the major communication difficulties that AA face is the constant feeling of being misunderstood (Table 2).^10^ Recent studies have found this kind of misunderstanding to be more complex than previously thought as the results indicate mutual misunderstandings between AA and non-AA individuals.^10^ This indicates that both AA and non-AA have difficulty in correctly interpreting the feelings and thought processes of the other neurotype.^10^ Interestingly, when AA communicate with other AA, their communication is just as effective as when non-AA communicate with other non-AA; the misunderstandings occur between AA and non-AA communications. These challenges can be explained by the double empathy problem, which suggests that people with markedly different social experiences—such as autistic and non-autistic individuals—have trouble empathizing with one another.^10^

**Table 2:** Common phrases to avoid when interacting with AA in STEMM

What Not to Say	Rationale	Instead, You Can Say
“You don’t look autistic.”	Unfortunately, a large proportion of AA have learned to hide or “mask” their symptoms to appear more neurotypical or “fit in” in society. Doing this is incredibly exhausting and can lead to even more feelings of isolation, anxiety, and depression as they are hiding their true self. Some autistics “unmask” when they are home or around people they trust who genuinely accept them. If this comment feels appropriate, you are probably not seeing an “unmasked” autistic person.	“Thank you for sharing that information with me.”
“I would have never known.”	This can feel like an invalidation of the unique experiences of AA.	
“We all have difficulty with (insert sensory issue).”	This invalidates the autistic adult’s struggle and the severity of their experience. Not everyone experiences sensory sensitivities to the same degree.	“Is there anything that I can do to help?”
“You seem normal to me.”	The person is a very normal autistic adult. It is important to consider there are different levels of autism.	“I hope that I can better educate myself about autism and the many different unique experiences that autistic individuals have.”
“Are you sure you’re autistic?”	AA are very aware of their differences from non-AA. Their experience is valid and does not require justification.	
“Don’t we all have a little autism?”	No. No, we do not. This invalidates the autistic adult’s struggle and the severity of their experience.	

##### Solutions

Given the heterogeneity among autistic individuals,^8^ there is no single fix for communication challenges. Many may benefit from and prefer honest, straightforward communication; these individuals can often find indirect communication to seem like a falsification of the truth, which increases feelings of isolation. We see a need for bidirectional training between autistic and non-autistic students, young adults, and working professionals, including employers and employees. Instead of outdated deficit-based training that places the burden of learning on AA, we believe that collaborative social skills groups that focus on reducing interpersonal miscommunications for both autistic and non-autistic individuals can be incredibly helpful.

Notably, among the general STEMM population, many individuals, approximately 50%, report difficulties in communicating their science to the general public.^11^ Thus, we believe it is important to begin to implement wider inclusive communication training, or spaces in the STEMM classroom to learn how to effectively communicate. This can be done with the implementation and introduction of the double empathy problem to specifically address and consider interpersonal communication with AA, as well as communication in general. For example, experts in leadership could lead talks about how to become leaders. Recently, training on STEMM communication has been developed through taking a multifaceted approach that teaches students through a combination of in-class techniques, virtual reality practice presentations, and social media-based communication.^12^ Beyond oral communication and presentation skills, participation in inclusive writing accountability groups can further bolster written communication skills, as well as give a casual environment to begin exploring networking, a skill often untaught in STEMM and likely needed the most by AA. It should be mentioned, however, that standardizing these interventions for all students can reduce potential marginalization felt by AA-specific pieces of training, while bolstering the wider scientific population’s communication and presentation skills.

#### Willingness to work with autistic adults

##### Challenge

The diversity within the autistic community may result in some individuals not wanting to work with AA. Moreover, though most people generally know the term autism, many do not understand what makes someone autistic. These differences are the reason autistic individuals require accommodations to be included in a society designed for non-autistic people. Even those with extensive education about autism may not know what it means to be autistic or have autistic people in their lives. Studies have indicated that organizations and employers who avoid or do not wish to hire AA are (1) uneducated about and unfamiliar with autism and (2) focused on AA’s limitations or potentially negative social behaviors rather than their strengths.^13^ These same employers failed to recognize the importance of providing support for autistic employees.^13^ This is a missed opportunity for both potential autistic employees and potential employers, especially in STEMM.

##### Solutions

Some organizations prioritize hiring AA because of their unique strengths, such as their attention to detail, reliability, trustworthiness, extreme focus, and willingness to do repetitive and socially isolating tasks, which are hallmarks of some STEMM fields.^6^^,^^13^ For example, image analyses^6^ benefit from the work of autistic specialists. If STEMM organizations are willing to modify their environment to include AA, both will reap the benefits. However, non-autistic people need to understand what it means to be autistic, including the different sensory and communication modifications necessary to be truly inclusive of AA in STEMM.

One excellent option for autism education at a systemic level in STEMM is mandatory autism humility training. We recommend that institutions implement this training across undergraduate studies as well as in the standard onboarding process for new faculty and staff. Humility training would be impactful in STEMM fields, especially as a replacement for autism competence training, which can give learners a false sense of expertise and tends to propagate stereotypes of the autistic community.^14^ Humility training allows participants to learn about autism from autistic individuals while gaining awareness of their biases, values, and beliefs.^14^ Many such trainings are already available for institutions (Table 3) or from offices of Diversity, Equality, and Inclusion (DEI). Autistic and non-AA should co-design the training protocols to reduce autism stigma. The importance of involving AA in autism humility training cannot be understated as it ensures that the education is informed by firsthand experience. It should also be noted that early education about neurodivergence can help cause a generational shift in mindfulness and allyship around autism, which will ultimately help institutions acquire and retain autistic individuals. Compassionate engagement from peers is a requisite, and everyone in STEMM, regardless of their position, may aid the inclusion of AA through such compassion.

**Table 3:** Additional resources/reading

Organization	About	Helpful Links
Autism Self Advocacy Network	Learn about autism from an autism non-profit organization ran by autistic people.	https://autisticadvocacy.org/about-asan/about-autism/
Job Accommodation Network	Free, expert, and confidential guidance for workplace accommodations. You can even see an A-to-Z list of disabilities and accommodations.	https://askjan.org/a-to-z.cfm https://askjan.org/toolkit/index.cfm
Microsoft: Neurodiversity Career Connector	A career marketplace for neurodiverse candidates.	https://ndcc.simplifyhire.com/
Neurodiversity @ Work Employer Roundtable	A collection of employers who seek neurodiverse talent to help their businesses thrive. Several STEMM-related employers participate.	https://disabilityin.org/what-we-do/committees/neurodiversity-at-work-roundtable/
Autism-Europe	International organization that offers numerous resources to autistic individuals, beyond advocacy, including employment opportunities and vocational support.	https://www.autismeurope.org
College Autism Network	An organization that promotes post-secondary outcomes, experience, and access for autistic individuals.	https://collegeautismnetwork.org/

Intentional mentorship is especially important in the context of training and supporting AA. Mentors of neurodiverse individuals should have mentor training, as well as approach mentorship with a sense of humility that avoids making assumptions and rather focuses on trainee growth. Equally so, mentors and principal investigators can further create inclusive environments by promoting accessibility at baseline for all, even if AA have not disclosed. This can include the accommodation options (Table 4) we have discussed being available in the laboratory, potentially before being requested when possible. Similarly, providing flexibility and understanding, in general, and especially in the event of involuntary sensory- or emotional-mediated meltdowns. Again, employers, supervisors, and principal investigators offering this general sense of flexibility and consideration is especially important for AA as knowing that this will be provided if necessary may reduce workplace anxiety.

**Table 4:** Sensory overstimulation considerations and possible accommodation options

Sensory & Other Considerations	Additional Information	Accommodation Options
Overload recovery management	Overloads occur when one or more senses take in more information than the brain can process, resulting in overstimulation. They can be very difficult to recover from. This is especially true if the autistic individual cannot recover properly away from excessive sensory stimuli.	• A quiet, relatively secluded space with adjustable lighting that is designated as a recovery area for those who have had a sensory overload.
Sound sensitivity management	Many autistic people are sensitive to sound; this includes the pitch, volume, and complexity of sounds. Although sound sensitivity severity can be different for each AA, for some, this can be a detrimental hindrance as it can trigger a sensory overload.	• Noise machine: brown and gray noise may work particularly well for autistic individuals. • Acoustic decoupling headphones. • Silent or reduced-noise computer keyboard and mouse. • A controlled and, if possible, more secluded work area.
Anxiety management	Many in the autistic population also have some form of anxiety disorder. This anxiety also ranges in severity between AA.	• Deep pressure sensory support: e.g., weighted, compression, or hug vests. Weighted lap pads might work as well. • Fidget toys. • Visual sensory stimulation: some autistic individuals find spinning objects or liquid motion bubblers calming.
Light sensitivity management	Some autistic individuals have sensory processing differences that manifest as hypersensitivity to certain types or intensities of light.	• Avoid or remove fluorescent and LED lighting. If possible, use only natural lighting. • Tinted glasses can help with sensitivity to fluorescent and LED lighting. • Light-blocking window shades may help if direct sunlight is an issue.
Executive function management	Executive function regulates an individual’s ability to plan, organize, monitor, and regulate their behavior. These functions are especially important for people in STEMM but also affect individuals’ personal lives.	• Utilize a calendar system that sends event reminders.
Auditory processing management	Auditory processing includes aspects of how autistic individuals perceive and interpret sounds and speech, which may be impacted by hypersensitivity to auditory stimuli.	• Allow people to record audio in meetings, while receiving directions, or in any situation when processing details is important. • Allow individuals additional time to process information, such as instructions, events, or questions. • Communication differences can make auditory processing more difficult. Encourage people to ask questions to clarify their understanding and interpretation of what was stated. Provide support for autistic individuals who ask for things to be restated or clarified due to marked verbal communication differences.
Handwriting and word processing management	Autistic individuals may have difficulties with fine motor skills and visual-motor speed, which can put them at a disadvantage in handwriting relative to their peers.	• Dictation software. • Allow individuals to use a laptop or word processor instead of handwriting. • Grip-assistive writing tools.

Beyond these specific accommodations, broader universal design principles should be adopted to further increase the inclusivity of all individuals with a disability at an institution.

#### Misconceptions and support

##### Challenge

There are several misconceptions in the non-autistic community about what it means to be autistic, and navigating these misconceptions can be especially isolating in the workplace. For instance, there is a more frequent depiction of autistic children in media compared with AA. However, autistic children become adults and as autistic children grow up, especially undiagnosed, they often learn how to “mask” their autistic traits to feel less alienated. Thus, although an autistic adult may appear “non-autistic” to the non-autistic people around them, their thought processes and perceptions of their surroundings are authentically autistic. Forgetting these differences, especially in those who mask their autistic traits well, often leads to misunderstandings and even severed relationships. As discussed under the subheading communication differences, the autistic use and interpretation of words, language, and the intent behind them differ significantly from most non-AA and even within groups of autistic individuals. Therefore, AA require additional but varying levels of support and understanding.

##### Solutions

When an autistic adult in STEMM requests an institution-approved accommodation, employers should ensure that they understand and support the request. Accommodations can take the form of modifications, such as a distraction-free environment, or assistive technology, such as notetaking software that records audio (Table 4). If an approved accommodation does not rectify the issue, an autistic adult can speak with their supervisor and/or the accessibility services office at their institution to find a solution. Accommodations may not automatically overcome the challenges many autistic individuals face in skills such as time management,^15^ but they can begin to reduce their burden. Importantly, accommodations can also provide an avenue for autistic individuals to begin advocating for their needs, and we believe that mentoring to help them do so will aid them in becoming self-advocates.

Providing and complying with accommodations for AA may yield better work output and higher retention rates in STEMM.^13^ Workspace environments that are inclusive of AA should avoid excessive sensory stimuli and distractions and allow for sufficient learning time, thus improving work output and perceived acceptance.^13^ The tasks assigned to AA should be defined clearly and, if possible, have a schedule that can be modified to fit within the individual’s preferred timeline.^13^ Furthermore, a shift toward open scholarship, which prioritizes quality over quantity in research, can reduce the current “publish-or-perish” culture which limits the success of many individuals within STEMM (see https://www.psychologicalscience.org/observer/gs-navigating-academia-as-neurodivergent-researchers).

Finally, social mindfulness can further improve inclusivity. Mindfulness is a consciousness of one’s experience in that moment, and social mindfulness extends this concept to incorporate outcome interdependence. We feel social mindfulness is especially important in STEMM environments (Table 4). For example, some autistic individuals can have a difficult time focusing on experiments or other work when there is background noise or excessive talking.^7^ AA may have subtle or drastic sensory differences, which can include both over- and under-sensitivity. The more socially mindful that the non-autistic population becomes of this heterogeneity, the more inclusive the environment will become for autistic people. The previously described bidirectional training programs or workshops designed by autistic individuals can thus increase social mindfulness among STEMM professionals.

### Conclusion

AA face considerable adversity in society today;^2^^,^^3^^,^^13^ however, conscious communication and environmental changes can alleviate their feelings of exclusion. Institutional changes informed by autistic people are needed to address barriers and create opportunities for all autistic individuals, regardless of their support needs. Autism is often approached from a deficit-based perspective,^6^^,^^7^^,^^8^^,^^15^ but it is important to consider how to foster the strengths of autistic individuals. For some AA, simple modifications to interactions and communication styles can help them feel included in STEMM. Others experience tremendous social, emotional, and cognitive difficulties that pose issues related to executive functioning and workplace knowledge.^7^ Increasingly, journals and social media have served as a platform for neurodivergent individuals, which serves as an important mechanism to showcase the diversity of neurodivergent experiences. We believe this is of paramount importance, and while we offer one such perspective, heterogeneity in STEMM experience is a defining aspect in neurodivergent individuals, and such diversity must continue to be highlighted.

Autistic individuals can become effective self-advocates to improve their chances of employment. In tandem, institutions must begin to dismantle the barriers that exclude autistic individuals. Still, the barriers are far more complex than discussed in this article, and we believe that removing them requires a multi-faceted approach involving social mindfulness, systemic- and professional-level changes, and bidirectional training, which may be promoted by numerous resource and advocacy agencies (Table 3). Though thriving in the workplace is often difficult for autistic individuals and requires mutual effort from both the employee and the employer,^7^ we aimed to highlight the unique perspectives and skills AA may offer, which are often neglected. Despite the rising prevalence of autism, autistic participation in STEMM may decrease rather than increasing in kind. Thus, education at a systemic level is critical to improving the general understanding of how to work with AA.
